# Outcomes for Surgery in Stage IA Large Cell Lung Neuroendocrine Compared With Other Types of Non-Small Cell Lung Cancer: A Propensity Score Matching Study Based on the Surveillance, Epidemiology, and End Results (SEER) Database

**DOI:** 10.3389/fonc.2020.572462

**Published:** 2020-11-26

**Authors:** Liqing Zou, Tiantian Guo, Luxi Ye, Yue Zhou, Li Chu, Xiao Chu, Jianjiao Ni, Zhengfei Zhu, Xi Yang

**Affiliations:** ^1^Department of Radiation Oncology, Fudan University Shanghai Cancer Center, Fudan University, Shanghai, China; ^2^Department of Oncology, Shanghai Medical College, Fudan University, Shanghai, China; ^3^Institute of Thoracic Oncology, Fudan University, Shanghai, China

**Keywords:** large cell lung neuroendocrine, stage IA, propensity score matching, SEER database, surgery

## Abstract

**Background:**

Pulmonary large cell neuroendocrine cancer (LCNEC) is commonly classified as non-small cell lung cancer (NSCLC). Even for stage I disease, after surgery the survival is always poor, but clinical research on LCNEC is scant and always with unsatisfying sample sizes. Thus, we conduct the first study using the Surveillance, Epidemiology, and End Results (SEER) database to compare survival after surgery between stage I LCNEC and other types of NSCLC.

**Methods:**

From 2004 to 2016, 473 patients with stage IA LCNEC, 17,669 patients with lung adenocarcinoma (LADC) and 8,475 patients with lung squamous cell cancer (LSCC), all treated with surgery were identified. In addition, 1:1 PSM was used, and overall (OS) and cancer-specific survival (CSS) between groups were compared.

**Results:**

The 5-year OS rates and CSS rates for LCNEC were 52.5% and 81.5%, respectively. Overall, both OS and CSS were significantly superior for stage IA LADC than LCNEC (for OS: HR 0.636, 95% CI 0.568-0.712; for CSS: HR 0.688, 95% CI 0.561–0.842, LCNEC as reference), while comparable for LSCC with LCNEC (for OS: HR 0.974, 95% CI 0.869–1.091; for CSS: HR 0.907, 95% CI 0.738–1.115). PSM generated 471 pairs when LCNEC was compared with LADC and both OS and CSS were significantly better in LADC than LCNEC (for OS: HR 0.580, 95% CI 0.491–0.686; for CSS: HR 0.602, 95% CI 0.446–0.814). Of note, for the subgroup of patients ≤ 65 years old, HRs for both OS and CSS were lower (for OS: HR 0.470; for CSS: HR 0.482). As for comparison between LCNEC and LSCC, PSM generated 470 pairs. Differently, only CSS was significantly superior in LSCC than LCNEC (HR 0.563, 95% CI 0.392–0.807), while OS was not. Further grouping by age showed only CSS between two groups for patients with age ≤ 65 years old was significantly different (P = 0.006).

**Conclusions:**

We report the first survival comparison after surgery between stage IA LCNEC and other types of NSCLC by SEER database and PSM. Our results demonstrated after surgery, stage IA LCNEC was worse in survival, especially compared to LADC. Extra clinical care should be paid, especially for younger patients. More studies investigating adjuvant therapy are warranted.

## Introduction

Large cell neuroendocrine cancer (LCNEC) of bronchus and lungs is a rare disease and constitutes approximately 3% in lung cancers (1). Although it is one of the four types of pulmonary neuroendocrine tumor (NET), different from other three NET [the typical, atypical carcinoid and small cell lung carcinoma (SCLC)], according to NCCN Guidelines [Version 2.2020, non-small cell lung cancer (NSCLC)], it is most commonly classified as a form of NSCLC (2, 3). Still, we cannot overlook that for LCNEC, there is indeed difference in its biological, clinical, and prognostic characteristics compared with other types of NSCLC [Lung squamous cell cancer (LSCC) and lung adenocarcinoma (LADC)]. As for the treatment of early-stage LCNEC, consistent with early-stage LSCC and LADC, surgery is recommended by guidelines. Even for stage I patients, however, after radical surgery the survival is always poor with a 5-year survival rate of 43%–67% (4–7). The confirmation of this is instrumental clinically. Yet, due to its low incidence, clinical researches on LCNEC are scant and always restricted to retrospective studies with unsatisfying sample sizes (7–10).

Supported by the National Cancer Institute (NCI), the Surveillance, Epidemiology, and End Results (SEER) Program covers clinical data from 18 different population-based cancer registries and covers 30% of the United States population (11). Thus, it provides valuable resources for rare cancer studies such as LCNEC. However, studies using the SEER database comparing survival in stage IA pulmonary LCNEC with other types of NSCLC have not been found. Although one retrospective study (12) using the National Cancer Database and a single-center retrospective study (13) of 125 LCNEC patients have been performed, inconsistent results lied on whether survival in early stage pulmonary LCNEC after surgery was comparable with other types of NSCLC. Larger-scale clinical data and methods to control bias brought by retrospective studies, such as propensity score matching (PSM) which can create matched set consisting of participants in the treatment group and control group with similar propensity scores, thus to approximate a random experiment by balancing covariates, are needed in further studies.

Based on above-mentioned clinical needs, we used the SEER database and PSM to conduct such survival comparison after surgery in stage IA (AJCC TNM-6:T_1_,N_0_,M_0_; AJCC TNM-7: T_1a_-T_1b_,N_0_,M_0_) NSCLC, and sought to investigate whether the survival difference after surgery exists for very early stage LCNEC.

## Patients and Methods

### Data Source and Ethics Statement

We used the specialized database “Incidence–SEER 9 Regs Custom Data (with additional treatment fields) of the SEER database Nov 2018 Sub (1975-2016) varying)” to extract data using the SEER*Stat software, version 8.3.6 (Released August 8, 2019). In this study, informed consent was not required, since identifying information of individual patients has been excluded. These data are available publicly and access to the SEER data was obtained by signing the SEER Research Data Agreement. Personal identifying information is not stored in the SEER database.

### Patient Selection

Patients from 2004 to 2016 were enrolled in the study. The inclusion criteria were as follows: (1) primary tumor in “Lung and Bronchus” [based on site recode ICD–O–3 (International Classification of Diseases for Oncology, Third Edition)/WHO 2008]; (2) histological confirmation of LCNEC (ICD–O–3 8012/3), LADC(ICD–O–3 8040/3) and LSCC (ICD–O–3 8070/3); (3) stage IA (AJCC TNM-6:T_1_,N_0_,M_0_; AJCC TNM-7: T_1a_-T_1b_,N_0_,M_0_); (4) received cancer-directed surgery; (5) complete demographic data and follow-up data. It is worth noticing that according to AJCC TNM-6, AJCC TNM-7, and even for AJCC TNM-8, stage IA NSCLC was all defined as tumor with a maximum diameter of 3cm, and with no involvement in visceral pleura, the main branches of the bronchus, lymph nodes and distant sites. Therefore, although recorded according to different versions of the staging, the patients we enrolled were with the same disease level and still represent the same groups of patients in current staging system (AJCC TNM-8).

### Statistical Analysis

Categorical variables were compared using Pearson’s chi-square tests. When accessing overall survival (OS), patients recorded as “Alive” were censored. When accessing cancer-specific survival (CSS), patients recorded as “Dead (missing/unknown cause of death)” and “Alive or dead of other cause” were censored. Due to potential differences among patients with stage IA LCNEC, LADC, and LSCC, PSM was used between LCNEC and LADC groups, and between LCNEC and LSCC groups. In short, a propensity score represents the probability that a unit with certain characteristics will be assigned to the treatment group and helps to balance multiple characteristics in retrospective studies to approximate a random experiment. In our study, variables included in the model were age at diagnosis, gender, race, location of primary sites, chemotherapy, and radiotherapy status, which were likely to have an impact on survival for patients with lung cancer according to previous researches (14–18). For PSM between LCNEC and LADC, and between LCNEC and LSCC, 1:1 PSM without replacement was implemented, and nearest neighbor matching method with caliber of 0.001 was used. Overall survival (OS) and CSS between groups were compared by the Kaplan–Meier method and log-rank test. Cox proportional hazard models were also used to estimate hazard ratio (HR) and 95% confidential interval (CI) between groups. Subgroup analyses were performed, and forest plots were created to better present each prognostic factor’s effect on OS and CSS. The patients were stratified to subgroups of different age (≤65 or >65 years), gender (male or female), race (black or white), pathological grade (well-moderate or poor-undifferentiated), location (upper lobe or lower lobe), radiotherapy (yes or no), chemotherapy (yes or no/unknown), and marital status (married or unmarried). Univariate Cox proportional hazard model was used to estimate the HRs. Multivariable Cox proportional hazards models were also performed. Statistical analyses and PSM were performed with SPSS (IBM SPSS Statistics 25.0, Chicago, IL, US). Statistical significance was considered if two-sided p-value < 0.05. Figures were generated by GraphPad Prism (GraphPad Software 6.01, Inc).

## Results

### Patient Characteristics

**Figure 1:**
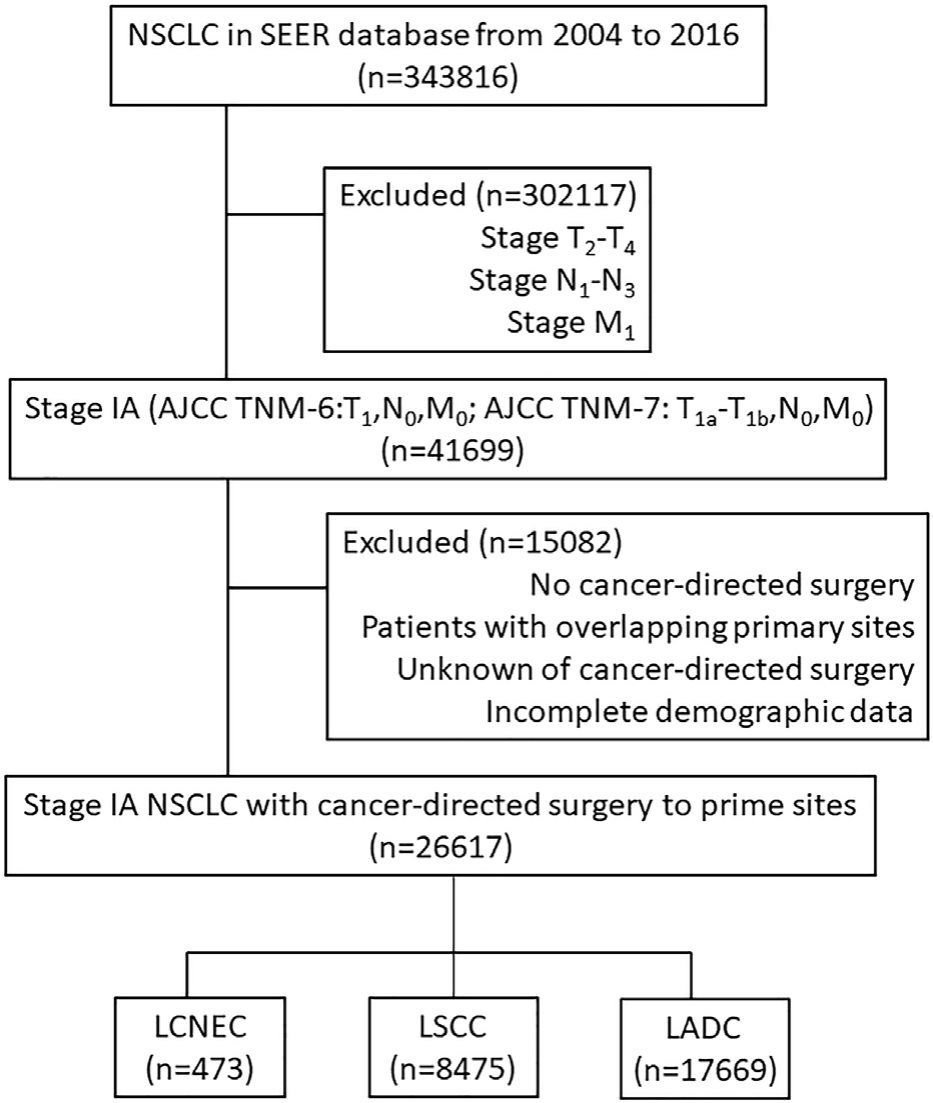
Flow diagram of enrollment. NSCLC, non-small cell lung cancer; the SEER database, Surveillance Epidemiology, and End Results database; AJCC, American Joint Committee on Cancer staging system; LCNEC, large cell neuroendocrine carcinoma; LSCC, lung squamous cell cancer; LADC, lung adenocarcinoma.

In summary, from 2004 to 2016, 343,816 patients were diagnosed with NSCLC, and 41699 of these patients were staged as IA (see **Figure 1** for flow diagram of enrollment). Of those patients, 26,617 documented to have received cancer-directed surgery also with complete demographic data and follow-up data were enrolled. Among them, 473 (1.8%) patients were diagnosed as LCNEC, 8,475 (31.8%) were diagnosed as LSCC, and 17,669 (66.4%) were diagnosed as LADC. Patient baseline demographics and pathological characteristics of all enrolled patients were listed and were compared among the three histologic types in **Table S1**.

### Survival Outcome Analysis Before PSM

We first compared survival among the three types of NSCLC before PSM. Both OS and CSS after surgery were significantly superior for patients with stage IA LADC (for OS: HR 0.636, 95% CI 0.568–0.712; for CSS: HR 0.688, 95% CI 0.561–0.842, LCNEC as reference, similarly hereinafter) than LCNEC, while comparable for LSCC (for OS: HR 0.974, 95% CI 0.869–1.091; for CSS: HR 0.907, 95% CI 0.738–1.115) with LCNEC (**Figure S1**). The 5-year OS rates for stage IA LCNEC, LADC, and LSCC were 52.5%, 66.6%, and 54.6%, respectively. The 5-year CSS rates for stage IA LCNEC, LADC, and LSCC were 81.5%, 86.9%, and 83.6%, respectively.

### Survival Outcome Analysis Between LCNEC and LADC After PSM

Patient baseline demographics and pathological characteristics of stage IA LCNEC and stage IA LADC before and after PSM were listed in **Table 1**. Stage IA LCNEC patients were more likely to be diagnosed at younger age (P < 0.001), with a higher proportion of male patients (P < 0.001), and more patients would undergo chemotherapy (P < 0.001) and radiotherapy (P < 0.001), compared to stage IA LADC. LCNEC was well matched with PSM generating 471 pairs and after PSM, these characteristics were well balanced.

**Table 1:** Patient baseline demographics and pathological characteristics of LCNEC and LADC before and after PSM.

Characteristic	Before PSM			After PSM		
	LCNEC (n = 473)	LADC (n= 17,669)	P value	LCNEC (n = 471)	LADC (n = 471)	P value
**Age at diagnosis**			**<0.001**			0.730
≤65	162 (34.2%)	3978 (22.5%)		160 (34.0%)	155 (32.9%)	
>65	311 (65.8%)	13,691 (77.5%)		311 (66.0%)	316 (67.1%)	
**Gender**			**<0.001**			0.794
Male	242 (51.2%)	7,343 (41.6%)		240 (51.0%)	236 (50.1%)	
Female	231 (48.8%)	10,326 (58.4%)		231 (49.0%)	235 (49.9%)	
**Race**			0.373			0.701
White	407 (86.0%)	15,011 (85.0%)		406 (86.2%)	400 (84.9%)	
Black	42 (8.9%)	1,478 (8.4%)		41 (8.7%)	41 (8.7%)	
other	24 (5.1%)	1,180 (6.7%)		24 (5.1%)	30 (6.4%)	
**Location**			0.646			0.450
Upper lobe	316 (66.8%)	11,434 (64.7%)		315 (66.9%)	321 (68.2%)	
Middle lobe	25 (5.3%)	966 (5.5%)		25 (5.3%)	32 (6.8%)	
Lower lobe	132 (27.9%)	5,269 (29.8%)		131 (27.8%)	118 (25.1%)	
**Chemotherapy**			**<0.001**			0.135
Yes	37 (7.8%)	659 (3.7%)		35 (7.4%)	48 (10.2%)	
No/Unknown	432 (92.2%)	17,010 (96.3%)		436 (92.6%)	423 (89.835%)	
**Radiotherapy**			**<0.001**			0.518
Yes	24 (5.1%)	411 (2.3%)		22 (4.7%)	18 (3.8%)	
No	449 (94.9%)	17,258 (97.7%)		449 (95.3%)	453 (96.2%)	

Bold values mean the difference is statistically significant.

**Figure 2:**
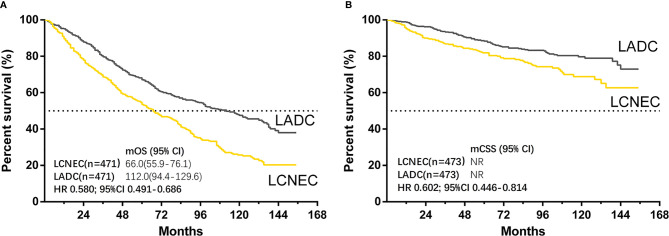
Kaplan–Meier curves for survival outcomes after PSM in LCNEC and LADC group: **(A)** overall survival (OS) and **(B)** cancer-specific survival (CSS) in matched patients between large cell neuroendocrine carcinoma (LCNEC) and lung adenocarcinoma (LADC) groups. CI, confidential interval; HR, hazard ratio; NR, not reached. LCNEC as reference.

The median follow-up time for matched cases was 55 months in LCNEC group and 91 months in LADC group. The results showed that both OS (LADC vs. LCNEC: median OS, 112.0 months vs. 66.0 months; HR 0.580; 95% CI 0.491–0.686; P < 0.001) and CSS (HR 0.602; 95% CI 0.446–0.814; P = 0.001) were significantly better in stage IA LADC group than in LCNEC group (**Figures 2A, B**). For matched LCNEC and LADC group, the 3- and 5-year OS rates were 67.3% vs. 80% and 52.5% vs. 66.8%, respectively; and 3- and 5-year CSS rates were 86.4% vs. 92.7% and 81.5% vs. 87.6%, respectively.

**Figure 3:**
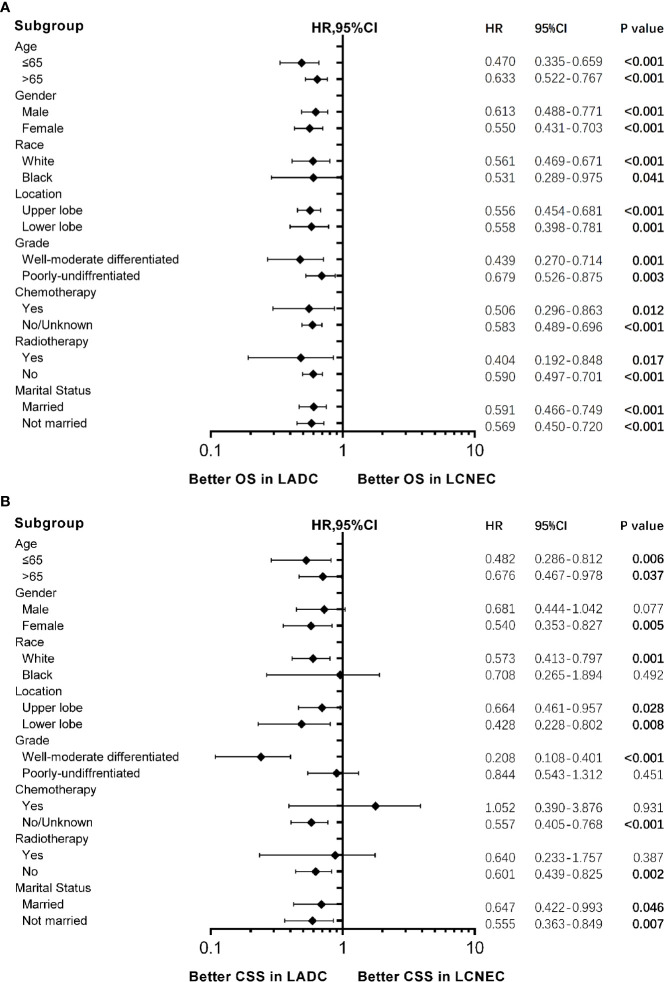
Forest plot of hazard ratios (HRs) for **(A)** overall survival (OS) and **(B)** cancer-specific survival (CSS) between stage IA large cell neuroendocrine carcinoma (LCNEC) and lung adenocarcinoma (LADC) in the subgroup analysis. The diamond on the X-axis indicates the HR and the 95% confident interval (CI) of each subgroup. LCNEC as reference.

Forest plots (**Figures 3A, B**) of HRs for OS and CSS were generated to illustrate subgroup analyses when stratifying patients by age, gender, race, location, chemotherapy, radiotherapy, and marital status. Consistent results showing stage IA LADC group was superior in OS than LCNEC were found in all subgroups (**Figure 3A**), and in CSS in most subgroups (**Figure 3B**). Of note, for patients in ≤65 subgroup, HRs for both OS and CSS were lower (for OS: HR 0.470, 95% CI 0.335–0.659, P < 0.001; for CSS: HR 0.482, 95% CI 0.281–0.812, P = 0.006).

To further investigate the factors influencing OS and CSS in these patients, a multivariate analysis was performed (**Table 2**). Consistent with prior results, histologic type was an independent factor for both OS and CSS (For OS, HR: 0.587, 95% CI: 0.452–0.762, P < 0.001; and for CSS, HR = 0.493, 95% CI: 0.314–0.776, P = 0.002). Intriguingly, we also noticed age was an independent factor for OS (P < 0.001) while not for CSS (P = 0.397). Thus, we also performed Kaplan-Meier analysis to show the influence of age on OS and CSS between matched groups (**Figure S2**), and the difference in OS and CSS was still significantly different between LCNEC and LADC in both ≤65 years old and >65-year-old subgroups.

**Table 2:** Multivariate analysis of overall survival (OS) and cancer-specific survival (CSS) predictors using cox proportional hazard model.

Characteristics	OS			CSS		
	HR	95%CI	P-value	HR	95%CI	P-value
**Age (yr)**			**<0.001**			0.397
≤65	Ref.			Ref.		
>65	1.844	1.519–2.239	**<0.001**	1.149	0.833–1.586	0.397
**Gender**			**<0.001**			0.321
Male	Ref.			Ref.		
Female	0.727	0.612–0.862	**<0.001**	0.856	0.630–1.164	
**Race**			**0.039**			0.743
White	Ref.			Ref.		
Black	0.734	0.534–1.010	0.058	0.838	0.493–1.424	0.513
Other	0.696	0.472–1.026	0.067	0.862	0.459–1.617	0.643
**Grade**			0.561			0.106
Well differentiated	Ref.			Ref.		
Moderate differentiated	1.212	0.876–1.675	0.246	1.681	0.891–3.172	0.109
Poorly differentiated	1.286	0.906–1.828	0.160	1.513	0.764–2.998	0.176
Undifferentiated	1.200	0.801–1.796	0.376	1.027	0.475–2.220	0.837
**Location**			0.105			0.217
Upper lobe	Ref.			Ref.		
Middle lobe	1.282	0.910–1.805	0.155	1.591	0.883–2.868	0.122
Lower lobe	0.877	0.722–1.065	0.184	0.908	0.638–1.292	0.592
**Chemotherapy**			**0.025**			0.655
No/unknown	Ref.			Ref.		
Yes	1.395	1.044–1.864	**0.025**	1.136	0.650–1.985	0.655
**Radiotherapy**			**<0.001**			**<0.001**
No	Ref.			Ref.		
Yes	2.432	1.694–3.492	**<0.001**	3.448	1.983–5.994	**<0.001**
**Histologic type**			**<0.001**			**0.002**
LCNEC	Ref.			Ref.		
LADC	0.587	0.452–0.762	**<0.001**	0.493	0.314–0.776	**0.002**
**Marital status**			**0.013**			0.297
Married	Ref.			Ref.		
Unmarried	1.243	1.047–1.476	**0.013**	1.178	0.866–1.603	0.297

Bold values mean the difference is statistically significant.

### Survival Outcome Analysis Between LCNEC and LSCC After PSM

Patient baseline demographics and pathological characteristics of stage IA LCNEC and stage IA LSCC before and after PSM were listed in **Table 3**. As was listed, stage IA LCNEC were more likely to be diagnosed at younger age (P < 0.001), with different races distribution (P = 0.027), and more patients would undergo chemotherapy (P < 0.001), compared to stage IA LSCC. LCNEC was well matched in PSM and PSM generated 470 pairs. After PSM, these different characteristics were well balanced.

**Table 3:** Patient baseline demographics and pathological characteristics of LCNEC and LSCC before and after PSM.

Characteristic	Before PSM			After PSM		
	LCNEC (n = 473)	LSCC (n = 8,475)	P value	LCNEC (n = 470)	LSCC (n = 470)	P value
**Age at diagnosis**			**<0.001**			0.579
≤65	162 (34.2%)	2274 (26.8%)		159 (33.8%0	151 (32.1%)	
>65	311 (65.8%)	6201 (73.2%)		311 (66.2%)	319 (67.9%)	
**Gender**			0.131			0.794
Male	242 (51.2%)	4637 (54.7%)		242 (51.5%)	247 (52.6%)	
Female	231 (48.8%)	3838 (45.3%)		228 (48.5%)	223 (47.4%)	
**Race**			**0.027**			0.444
White	407 (86.0%)	7592 (89.6%)		407 (86.6%)	396(84.3%)	
Black	42 (8.9%)	614 (7.2%)		39 (8.3%)	41 (8.7%)	
other	24 (5.1%)	269 (3.2%)		24 (5.1%)	33 (7.0%)	
**Location**			0.121			0.485
Upper lobe	316 (66.8%)	5,326 (62.8%)		315 (67.0%)	308 (65.5%)	
Middle lobe	25 (5.3%)	402 (4.7%)		25 (5.3%)	19 (4.0%)	
Lower lobe	132 (27.9%)	2747 (32.4%)		130 (27.7%)	143 (30.4%)	
**Chemotherapy**			**<0.001**			1.000
Yes	37 (7.8%)	304 (3.6%)		34 (7.2%)	34 (7.2%)	
No/Unknown	436 (92.2%)	8,171 (96.4%)		436 (92.8%)	436 (92.8%)	
**Radiation**			0.064			0.881
Yes	24 (5.1%)	293 (3.5%)		23 (4.9%)	24 (5.1%)	
No	449 (94.9%)	8182 (96.5%)		447 (95.1%)	446 (94.9%)	

Bold values mean the difference is statistically significant.

**Figure 4:**
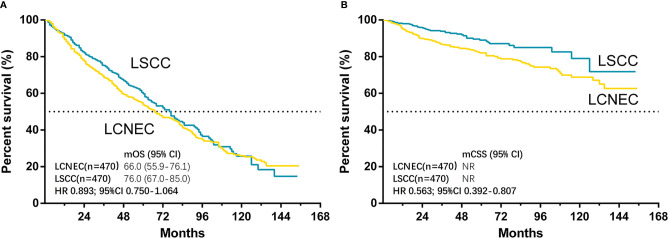
Kaplan–Meier curves for survival outcomes after PSM in LCNEC and LSCC group: **(A)** overall survival (OS) and **(B)** cancer-specific survival (CSS) in matched patients between large cell neuroendocrine carcinoma (LCNEC) and lung squamous cell cancer (LSCC) groups. CI, confidential interval; HR, hazard ratio; NR, not reached. LCNEC as reference.

**Figure 5:**
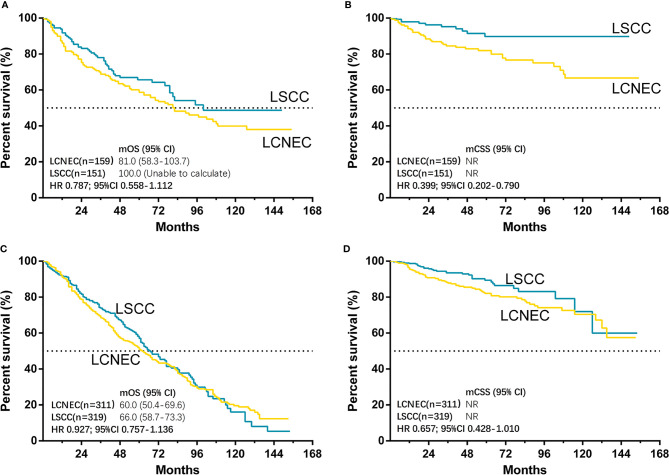
Kaplan–Meier curves for survival outcomes after PSM in LCNEC and LSCC ≤65 years old and >65 years old subgroups: **(A)** overall survival (OS) and **(B)** cancer-specific survival (CSS) in matched patients between large cell neuroendocrine carcinoma (LCNEC) and lung squamous cell cancer (LSCC) ≤65 years old subgroup; and **(C)** OS and **(D)** CSS in >65 years old subgroups. CI, confidential interval; HR, hazard ratio; NR, not reached. LCNEC as reference.

The median follow-up time for matched cases was 55 months in LCNEC group and 45 months in LSCC group. The results showed that only CSS was significantly better in LSCC group than LCNEC group (HR, 0.563; 95% CI, 0.392–0.807; P = 0.002), while OS was not (HR, 0.893; 95% CI, 0.750–1.064; P = 0.204) (**Figures 4A, B**). For matched LCNEC and LSCC group, the 3- and 5-year OS rates were 67.1% vs. 74.2% and 52.4% vs. 58.8%; and 3- and 5-year CSS rates were 86.3% vs. 93.2% and 81.7% vs. 88.4%, respectively. We further grouped by age (**Figure 5**) and for patients with age ≤ 65, only CSS between two groups was significantly different (P = 0.006). In patients with age ≤ 65, there was a trend (P = 0.171) showing OS of LSCC was superior, and Kaplan-Meier analysis for OS also showed disjoint curves, however, the difference was not significant. In elder patients, there was also a trend (P = 0.054) that in patients with age > 65, CSS of LSCC was superior around before 120 months and the curve intersected. Still, these results reflected for stage IA LCNEC and LSCC patients after surgery to primary site, LSCC was superior in survival than LCNEC, while the difference was moderate.

**Figure 6:**
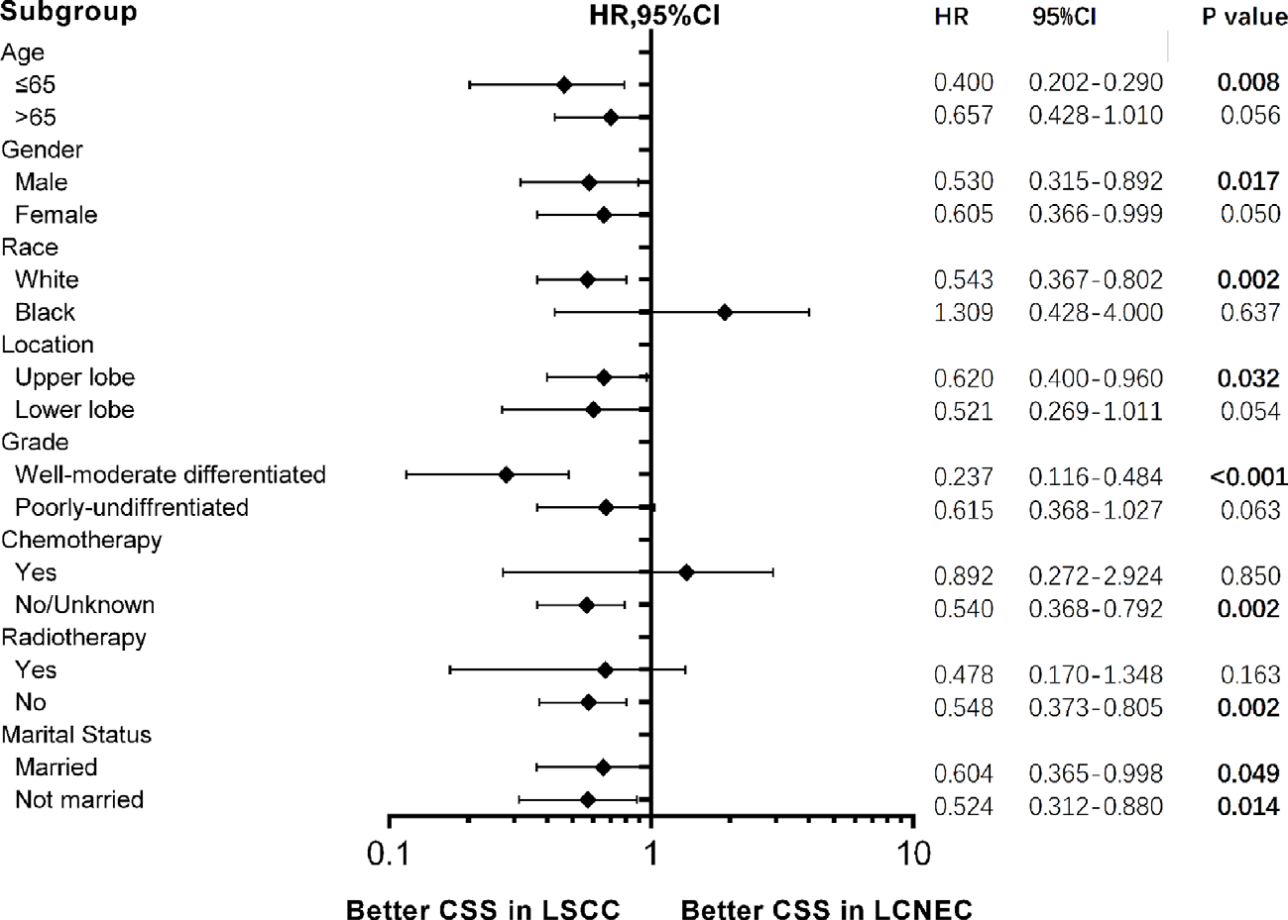
Forest plot of hazard ratios (HRs) for cancer-specific survival (CSS) between stage IA large cell neuroendocrine carcinoma (LCNEC) and lung squamous cell cancer (LSCC) in the subgroup analysis. The diamond on the X-axis indicates the HR and the 95% confident interval (CI) of each subgroup. LCNEC as reference.

Forest plots of HRs for OS (**Figure S3**) and CSS (**Figure 6**) was generated to illustrate the exploratory subgroup analyses when stratifying patients by age, gender, race, location, chemotherapy, radiotherapy, and marital status. Results showing stage IA LSCC group was superior in CSS than LCNEC were found in most subgroups, but no significant difference was found in age > 65 years old, black, female, lower lobe, poorly-undifferentiated, radiotherapy, and chemotherapy subgroups. It is also worth noticing that in subgroup analysis for patients with age≤65, a lower HR for CSS was observed (HR 0.400, 95CI 0.202–0.290, P = 0.008).

Multivariate analysis was performed to further investigate the factors influencing OS and CSS in matched patients (**Table 4**). On multivariate analysis, consistent with prior results, histologic type was an independent factor for CSS (HR: 0.404, 95% CI: 0.248–0.659, P < 0.001), but not for OS (HR: 0.924, 95% CI: 0.724–1.181, P = 0.530). Also, similar to the comparison with LADC, age was an independent factor for OS (HR: 1.587, 95% CI: 1.301-1.935, P < 0.001) but not for CSS (HR: 1.079, 95% CI: 0.751–1.551, P = 0.679).

**Table 4:** Multivariate analysis of overall survival (OS) and cancer-specific survival (CSS) predictors using cox proportional hazard model.

Characteristics	OS			CSS		
	HR	95%CI	P-value	HR	95%CI	P-value
**Age (yr)**			**<0.001**			0.679
≤65	Ref.			Ref.		
>65	1.587	1.301–1.935	**<0.001**	1.079	0.751–1.551	0.679
**Gender**			**<0.001**			0.971
Male	Ref.			Ref.		
Female	0.715	0.598–0.856	**<0.001**	1.007	0.710–1.427	0.971
**Race**			**0.015**			0.403
White	Ref.			Ref.		
Black	0.852	0.626–1.160	0.308	0.893	0.499–1.600	0.704
Other	0.553	0.364–0.839	**0.005**	0.570	0.247–1.315	0.188
**Grade**			0.490			0.219
Well differentiated	Ref.			Ref.		
Moderate differentiated	0.929	0.453–1.904	0.840	0.777	0.2327–2.554	0.678
Poorly differentiated	1.126	0.553–2.291	0.890	0.612	0.189–1.984	0.413
Undifferentiated	1.204	0.869–1.670	0.265	0.439	0.131–1.475	0.183
**Chemotherapy**			0.265			0.911
No/Unknown	Ref.			Ref.		
Yes	1.204	0.869–1.670	0.265	0.964	0.503–1.846	0.911
**Radiotherapy**			**<0.001**			**<0.001**
No	Ref.			Ref.		
Yes	2.198	1.560–3.097	**<0.001**	3.407	1.921–6.042	**<0.001**
**Histologic type**			0.530			**<0.001**
LCNEC	Ref.			Ref.		
LSCC	0.924	0.724–1.181	0.530	0.404	0.248–0.659	**<0.001**
**Marital status**			**0.005**			0.393
Married	Ref.			Ref.		
Unmarried	1.290	1.079–1.543	**0.005**	1.164	0.821–1.651	0.393

## Discussion

LCNEC of bronchus and lungs is a rare disease and is one of the four types of pulmonary NETs, according to NCCN Guidelines (Version 2.2020, NSCLC). Like early stage LSCC and LADC, surgery is recommended by guidelines. However, in our study for stage IA LCNEC, the survival after radical surgery is still poor with a 5-year OS rate of 52.5%, consistent with the 5-year survival rate of 43%–67% in previous studies (4–7). By comparison in our research, both OS and CSS after surgery were significantly superior for patients with stage IA LADC than LCNEC (for OS: HR 0.636; for CSS: HR 0.688), while comparable for LSCC with LCNEC (for OS: HR 0.974; for CSS: HR 0.907). The 5-year OS rate for stage IA LCNEC, LADC, and LSCC were 52.5%, 66.6%, and 54.6%, respectively. The survival data was basically consistent with previous study (12). Slightly different from our study, the results of previous study (12) indicated stage I LCNEC was worse in OS after surgery compared with LADC and LSCC.

To better control the bias brought by retrospective clinical data, PSM was used to create a highly comparable control group. PSM generated 471 pairs when LCNEC group was compared with LADC group, and both OS (HR, 1.724) and CSS (HR, 1.661) were significantly worse in stage IA LCNEC group than in LADC group. Consistent results showing stage IA LADC group was superior for OS than LCNEC were found in all subgroups and for CSS in most subgroups. Of note, for the subgroup of patients ≤ 65 years of age, HRs for both OS and CSS were lower (for OS: HR, 0.470; for CSS: HR, 0.482). On multivariate analysis, consistent with prior results, histologic type was an independent factor for both OS and CSS. As for comparison between LCNEC and LSCC, PSM generated 470 pairs. Only CSS was significantly better in LSCC than LCNEC (HR, 0.563), while OS was not. Further grouping by age showed only CSS between two groups for patients with age≤65 years old was significantly different (P = 0.006), and in subgroup analysis for patients with age ≤ 65, a lower HR for CSS was observed (HR, 0.400). Collectively, these results demonstrated stage IA NSCLC was worse in survival, especially than LADC. Clinically special attention should be paid to younger patients with LCNEC, because they showed significantly worse survival compared to other types of NSCLC.

Thus, even for disease at extremely early stage, besides radical surgery, more clinical studies investigating whether adjuvant therapy should also be considered in treatment modality are warranted. Although available literature regarding the treatment of early-stage LCNEC is scant, current evidence indicates adjuvant or neo-adjuvant chemotherapy may play a major role (19). On the one hand, previous small sample analysis indicated adjuvant chemotherapy may bring survival benefits. In a multicenter retrospective analysis (7), 144 patients with LCNEC who underwent lung resection were reviewed, and in stage I (n = 73) disease, adjuvant or induction chemotherapy had a trend (P = 0.077) showing better outcome compared with no chemotherapy. Another retrospective study (9) reviewed 45 patients with LCNEC who underwent surgery, and it also showed despite the small sample size, the survival benefit of adjuvant chemotherapy can still be observed even in the stage I LCNEC cases [surgery plus adjuvant chemotherapy (n = 11), surgery alone (n = 16)]. On the other hand, in terms of treatment regimen, there are also several other prospective (20) and retrospective studies (8, 21–25) showing platinum-based (usually cisplatin + etoposide or irinotecan) chemotherapy plus surgery achieved better survival than surgery alone. Also, a randomized phase III trial (26) is now ongoing in Japan to compare the adjuvant cisplatin + etoposide regimen with cisplatin + irinotecan regimen in patients with completely resected high-grade LCNEC.

This study had certain limitations. First, although our study also showed better survival in stage IA LSCC than LCNEC, unlike previous analysis (12), the difference in our study was only significant in some subgroups, perhaps attributed to our smaller sample size and larger bias of previous study. Larger scale data with smaller bias are called for. Still, both studies consistently emphasize extra attention should be paid to LCNEC compared to other early-stage NSCLC. Second, although PSM was performed, the analyses are essentially retrospective and it inevitably comes with a selection bias. Finally, details of chemotherapy and radiotherapy are limited in the SEER registry and it is not prudent to address the specific effect of the addition of chemotherapy or radiotherapy based on current data, thus current chemotherapy and radiotherapy data were only used for PSM but cannot be used to address whether the adjuvant therapy can achieve better survival than surgery alone. In the design of the experiment, we also assumed that the number of lymph nodes dissected may have an impact on the prognosis. SEER did record the number of local lymph nodes dissected and the number of lymph nodes biopsied. However, in the number of lymph nodes resected, half of the patients were recorded none or unknown; also, in the number of lymph nodes biopsied, half of the patients were recorded as no lymph nodes was examined, or although the examination was performed but the specific number was not recorded, or status of lymph nodes biopsied was not recorded in the medical history. These made us think that this part of the data is not convincing enough to analyze. Despite these limitations, information that plays a vital role in the PSM was available and analyzed in this study.

In conclusion, we report the first survival comparison between stage IA LCNEC and other types of NSCLC patients who received surgery from data of the SEER database by using PSM analysis. Our results demonstrated that after surgery, stage IA LCNEC was worse in survival than LADC and LSCC, especially compared with LADC. Even for disease at extremely early stage, extra clinical care should be paid compared with other types of NSCLC, and besides radical surgery, especially for younger patients, more clinical studies investigating whether adjuvant therapy should also be considered in treatment modality are warranted.

## Data Availability Statement

Publicly available datasets were analyzed in this study. These data can be found at the Surveillance, Epidemiology, and End Results (SEER) database.

## Author Contributions

LZ, TG, and LY contributed equally to this work. All authors contributed to the article and approved the submitted version.

## Funding

This work was supported by National Natural Science Foundation of China (No. 81872461, 81703024).

## Conflict of Interest

The authors declare that the research was conducted in the absence of any commercial or financial relationships that could be construed as a potential conflict of interest.
