# Supplementary material for: Outcomes for Surgery in Stage IA Large Cell Lung Neuroendocrine Compared With Other Types of Non-Small Cell Lung Cancer: A Propensity Score Matching Study Based on the Surveillance, Epidemiology, and End Results (SEER) Database
Source: Front Oncol. 2020 Nov 26;10:572462. doi: 10.3389/fonc.2020.572462 (PMC7727448; doi:10.3389/fonc.2020.572462)
Supplement: Supplementary file 1 [file DataSheet_1.pdf]

Table S1. Patient baseline demographics and pathological characteristics of full sample.

Characteristics	LCNEC(n=473)	LSCC(n=8475)	LADC(n=17669)
**Age (yr)**			
≤65	162(34.2%)	2274(26.8%)	3978(22.5%)
> 65	311(65.8%)	6201(73.2%)	13691(77.5%)
**Gender**			
Male	242(51.2%)	4637(54.7%)	7343(41.6%)
Female	231(48.8%)	3838(45.3%)	10326(58.4%)
**Race**			
White	407(86.0%)	7592(89.6%)	15011(85.0%)
Black	42(8.9%)	614(7.2%)	1478(8.3%)
Other	24(5.1%)	269(3.2%)	1180(6.7%)
**Grade**			
Well differentiated	2(0.4%)	309(3.7%)	4266(24.1%)
Moderate differentiated	23（4.9%)	4512(53.2%)	8987(50.9%)
Poorly differentiated	203(42.9%)	3613(42.6%)	4326(24.5%)
Undifferentiated	245(51.8%)	41(0.5%)	90(0.5%)
**Chemotherapy**			
Yes	37(7.8%)	304(3.6%)	659(3.7%)
No/Unknown	436(92.2%)	8171(96.4%)	17010(96.3%)
**Radiotherapy**			
Yes	24(5.1%)	293(3.5%)	411(2.3%)
No	449(94.9%)	8182(96.5%)	17258(97.7%)
**Location**			
Upper lobe	316(66.8%)	5326(62.8%)	11434(64.7%)
Middle lobe	25(5.3%)	402(4.8%)	966(5.5%)
Lower lobe	132(27.9%)	2747(32.4%)	5269(29.8%)
**Marital status**			
Married	253(53.5%)	4677(55.2%)	9970(56.1%)
Unmarried	220(46.5%)	3798(44.8%)	7749(43.9%)

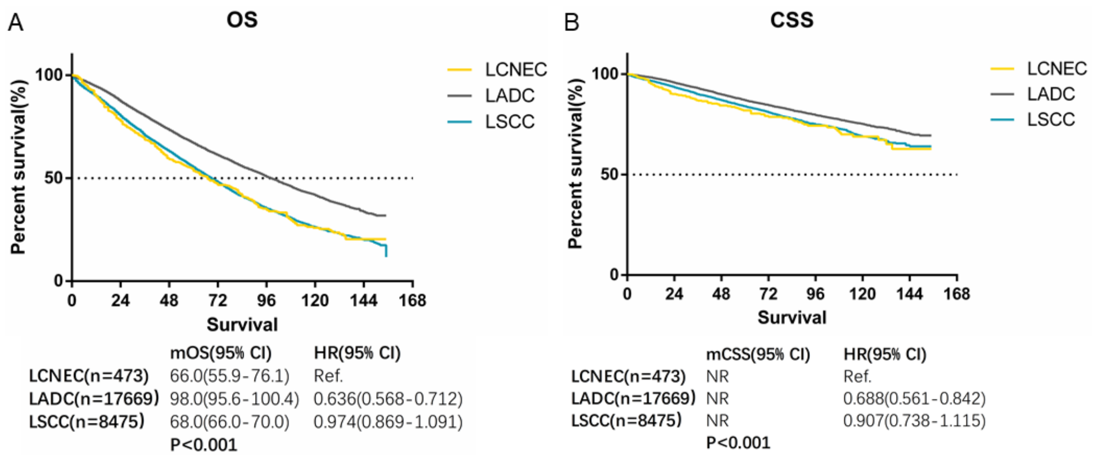

Figure S1. Kaplan–Meier curves for survival outcomes before PSM: (A) overall survival (OS) and (B) cancer-specific survival (CSS) in patients before PSM among all enrolled patients with large cell neuroendocrine carcinoma (LCNEC), lung adenocarcinoma (LADC), and lung squamous cell cancer (LSCC). CI, confidential interval. HR, hazard ratio. NR, not reached. Ref, reference.

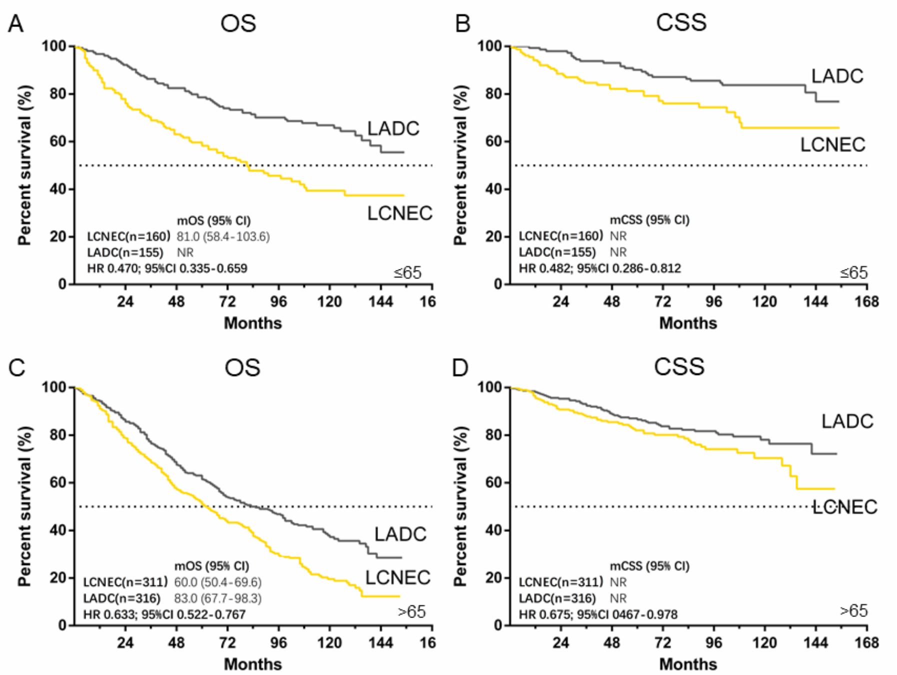

Figure S2. Kaplan–Meier curves for survival outcomes after PSM in LCNEC and LADC ≤65 years old and >65 years old subgroups: (A) overall survival (OS) and (B) cancer-specific survival (CSS) in matched patients between large cell neuroendocrine carcinoma (LCNEC) and lung adenocarcinoma (LADC) ≤65 years old subgroup; and (C) OS and (D) CSS in > 65 years old subgroups. CI, confidential interval. HR, hazard ratio. NR, not reached. LCNEC as reference.

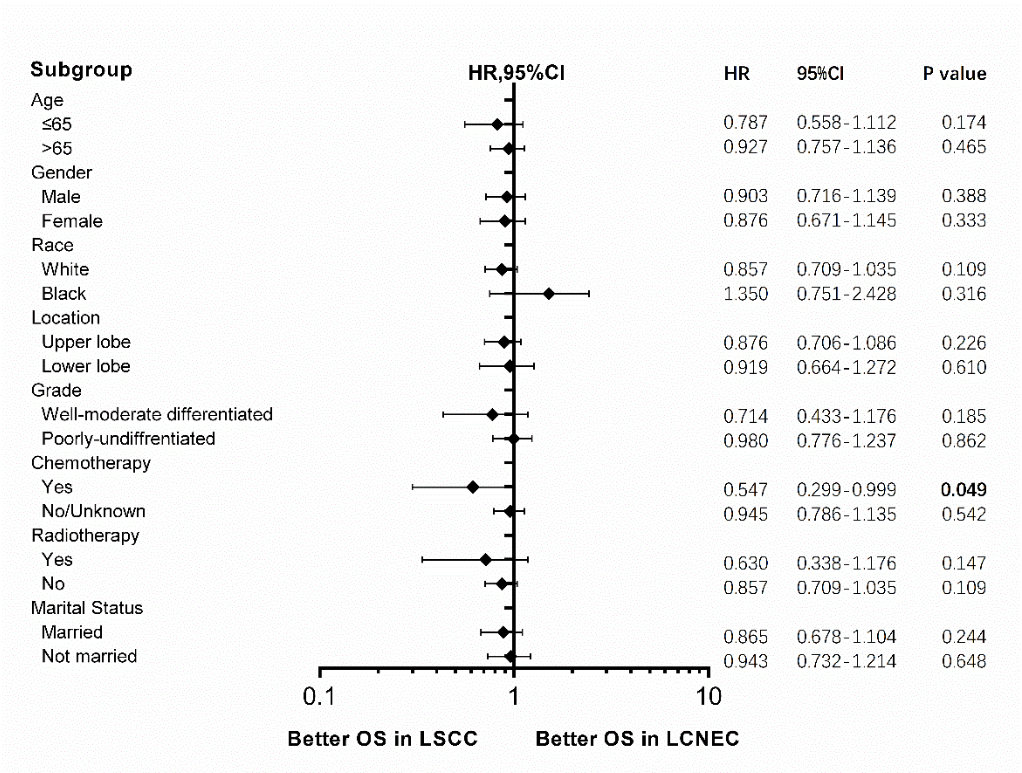

Figure S3. Forest plot of hazard ratios (HRs) for (A) overall survival (OS) and (B) cancer-specific survival (CSS) between stage IA large cell neuroendocrine carcinoma (LCNEC) and lung squamous cell cancer (LSCC) in the subgroup analysis. The diamond on the X-axis indicates the HR and the 95% confident interval (CI) of each subgroup. LCNEC as reference.
